# Escape and surveillance asymmetries in locusts exposed to a Guinea fowl-mimicking robot predator

**DOI:** 10.1038/s41598-017-12941-z

**Published:** 2017-10-09

**Authors:** Donato Romano, Giovanni Benelli, Cesare Stefanini

**Affiliations:** 1 The BioRobotics Institute, Scuola Superiore Sant’Anna, Viale Rinaldo Piaggio 34, 56025 Pontedera, Pisa, Italy; 2 Department of Agriculture, Food and Environment, University of Pisa, Via del Borghetto 80, 56124 Pisa, Italy; 3 Department of Biomedical Engineering and Robotics Institute, Khalifa University PO Box, 127788 Abu Dhabi, UAE

## Abstract

Escape and surveillance responses to predators are lateralized in several vertebrate species. However, little is known on the laterality of escapes and predator surveillance in arthropods. In this study, we investigated the lateralization of escape and surveillance responses in young instars and adults of *Locusta migratoria* during biomimetic interactions with a robot-predator inspired to the Guinea fowl, *Numida meleagris*. Results showed individual-level lateralization in the jumping escape of locusts exposed to the robot-predator attack. The laterality of this response was higher in *L. migratoria* adults over young instars. Furthermore, population-level lateralization of predator surveillance was found testing both *L. migratoria* adults and young instars; locusts used the right compound eye to oversee the robot-predator. Right-biased individuals were more stationary over left-biased ones during surveillance of the robot-predator. Individual-level lateralization could avoid predictability during the jumping escape. Population-level lateralization may improve coordination in the swarm during specific group tasks such as predator surveillance. To the best of our knowledge, this is the first report of lateralized predator-prey interactions in insects. Our findings outline the possibility of using biomimetic robots to study predator-prey interaction, avoiding the use of real predators, thus achieving standardized experimental conditions to investigate complex and flexible behaviours.

## Introduction

Predator-prey interactions are processes influencing the distribution, abundance and dynamics of animal populations in the ecosystems^1–3^. In addition, they are key selective mechanisms affecting both morphological and behavioural features in the animal kingdom^4^. There is a range of evidences from vertebrates demonstrating lateralized responses to predators^5,6^; as a general trend, they show more reactive behaviours to predators when they are seen by the left eye over the right one^7–10^, probably because the specialization of one hemisphere of the brain for a particular function allows the other hemisphere to perform different functions^9^.

Lateralization (i.e., left-right asymmetries in the brain and behaviour) has been reported in all vertebrate classes concerning different behaviours^9,11–14^. It offers critical advantages, such as increasing brain efficiency in cognitive tasks by avoiding duplications of functions in the two hemispheres^9,15^, and processing several streams of information in parallel^15,16^. However, lateralization could produce some disadvantages in the case of a prey reactive behaviour to a predator, as a lateral biased response can be predicted by predators^9^.

In insects, asymmetrical responses to predators have not been investigated, although recently, several studies reported other lateralized traits in different insect orders^15,17–28^. Acrididae use cryptic colorations and/or behavioural traits to go unnoticed during predator scanning^29–31^. They avoid predatory attacks by using their powerful hind legs, well adapted for jumping^32^. Thus, they are valuable models to test lateralized responses to predators in insects. When locusts are approached by a predator (e.g., an avian insectivore^33,34^), trains of spikes are produced by a bilateral pair of visual neurons (the lobula giant movement detectors, LGMDs), that are transmitted to the descending contralateral movement detector (DCMD), and then to the thorax motor centres, triggering the jump^35,36^. However, in locusts the jump trajectory is controlled by foreleg movements that produce rapid rolling and yawing of their body, thus they exhibit their trajectory decision after their hind legs have been cocked for jumping^32^. Interestingly, the desert locust *Schistocerca gregaria* (Forskål) (Orthoptera: Acrididae) was found lateralized at individual level in its forelimb use^37^. Strongly biased locusts made fewer errors with their preferred forelimb, suggesting that stronger lateralization provides an advantage in terms of boosted motor control in an invertebrate with individual-level lateralization^37^. These inferences suggested us to investigate the presence of lateral bias in Acrididae during predator-prey interactions.

In this study, to simulate a predation event, we developed a robot predator inspired to the helmeted Guinea fowl, *Numida meleagris* (Linnaeus) (Galliformes: Numididae), a natural enemy of locusts, used as a biological control agent in several regions^38–40^. This engineered approach, merging robotics with ethology, also known as ethorobotics^41–43^, allows us to produce readily controllable life-like stimuli that can allow more realistic interactions with animals^44,45^ if compared to traditional static dummies and 2-dimensional video-playbacks^46–49^. Furthermore, bioinspired engineers, aiming to unravel the exceptional strategies adopted by animals to reproduce them in advanced robots^50–54^, could profit from this scientific field merging natural and artificial agents in the same system and thus offering novel paradigms to design bioinspired artefacts. Recently, a growing number of studies employed robots for behavioural investigations^42,44,45,55–60^. Although one of the earlier use of robots to study insects dates to 1989^61^, only few examples are available about insect behavioural research^62–66^.

In this research, lateralized escape and predator surveillance behaviours in neanids, nymphs and adults of *Locusta migratoria* Linnaeus (Orthoptera: Acrididae) at gregarious phase^67^, were investigated at individual and at population-level during interactions with a Guinea fowl-mimicking robotic predator. It has been proposed that lateralization at population-level is more likely to evolve in social/gregarious species^9,68^, although lateralization at population-level has been reported in several solitary species^21,24,27^. We performed two different experiments to evaluate: (*i*) if *L. migratoria* shows any lateral bias when – startled by an approaching predator – it jumps off, and (*ii*) if there is an eye preference used for overseeing a potentially threatening animal. In addition, we investigated if these lateralized responses to a predator change varied among the different developmental stages, since in insects post-embryonic development affects numerous morphological, physiological and behavioural features^69–72^.

## Results

### Experiment 1: laterality of escape responses in locusts

The jumping escape response to a Guinea fowl-mimicking robotic predator was lateralized at the individual level, while the same was not true at the population level (*F*_*2,89*_ = 0.312; *P* < 0.7330). Right-biased adult locusts were not significantly more abundant than left-biased adult locusts, the same was true for IV and II instars.

**Figure 1:**
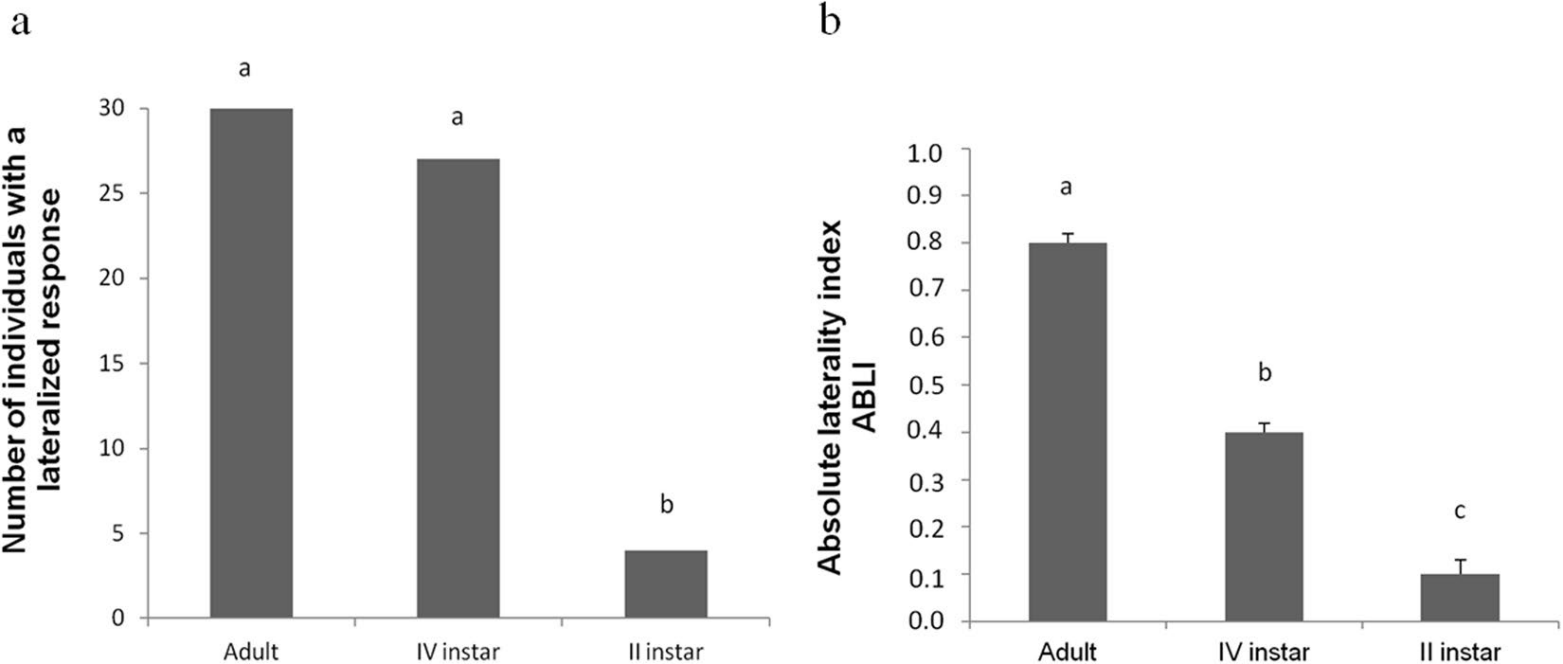
(**a**) *Locusta migratoria* individuals that exhibit a lateralized jumping escape response at individual level during the exposure to a Guinea fowl-mimicking robotic predator. Different letters above each column indicate significant differences. (**b**) Absolute value of ABLI for the jumping escape response of *L. migratoria* during the exposure to a Guinea fowl-mimicking robotic predator. Different letters above each column indicate significant differences. T-bars represent standard errors.

The presence of a jumping escape response at the individual level (regardless the left or the right direction), was significantly affected by the insect instar (*F*_*2,89*_ = 95.151; *P* < 0.0001). It was higher in adults and IV instar locusts over II instar ones (Fig. 1a).

The absolute values of the laterality index (ABLI) were significantly influenced by the locust instar (*F*_*2,89*_ = 207.404; *P* < 0.0001). ABLI in adults was significantly stronger over ABLI in IV instar locusts; ABLI in IV instars was stronger than the one calculated for II instars (Fig. 1b).

### Experiment 2: laterality of predator surveillance in locusts

**Figure 2:**
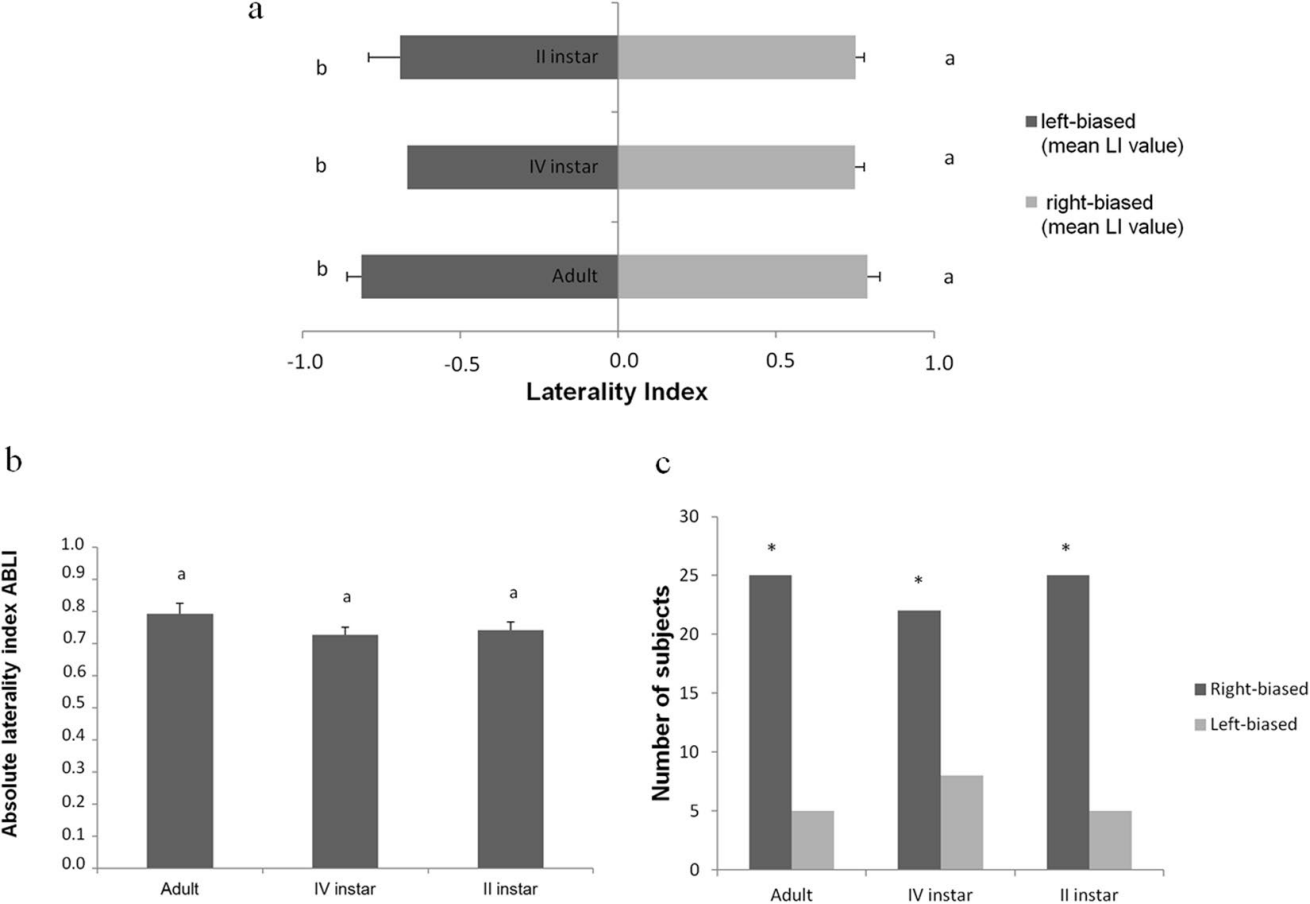
(**a**) LI values for *Locusta migratoria* surveillance during the exposure to a Guinea fowl-mimicking robotic predator. Different letters near each bar indicate significant differences. T-bars represent standard errors. (**b**) Absolute value of ABLI for the eye use in *L. migratoria* surveillance during the exposure to a Guinea fowl-mimicking robot. Different letters above each column indicate significant differences. T-bars represent standard errors. (**c**) Left- and right-biased use of eyes in *L. migratoria* surveillance during the exposure to a Guinea fowl-mimicking robotic predator. Asterisks indicate significant differences between left- and right-biased subjects.

*Locusta migratoria* adults and young instars showed lateralized eye use during the surveillance of the Guinea fowl-mimicking robotic predator. The laterality index (LI) value significantly differed between left- and right-biased individuals (*F*_*1,84*_ = 1319.947; *P* < 0.0001). No significant differences were detected among adult, IV instar and II instar locusts showing the same lateral bias (Fig. 2a). The absolute value of ABLI concerning the use of a compound eye for predator surveillance did not differ among tested instars (*F*_*2,87*_ = 1.561; *P* = 0.216). A right-biased lateral dominance was observed in adult, IV instar and II instar locusts (Fig. 2b).

Lateralized eye use for predator surveillance was noted at population-level by adult, IV instar and II instar locusts. Adult locusts preferentially use the right eye over the left one for predator surveillance (right vs. left: 25 vs. 5; *χ* ^*2*^_*1*_ = 52.61; *P* < 0.0001). The same was true for IV instars (right vs. left: 22 vs. 8; *χ* ^*2*^_*1*_ = 50.69; *P* < 0.0001) and II instars (right vs. left: 25 vs. 5; *χ* ^*2*^_*1*_ = 52.61; *P* < 0.0001), (Fig. 2c).

**Figure 3:**
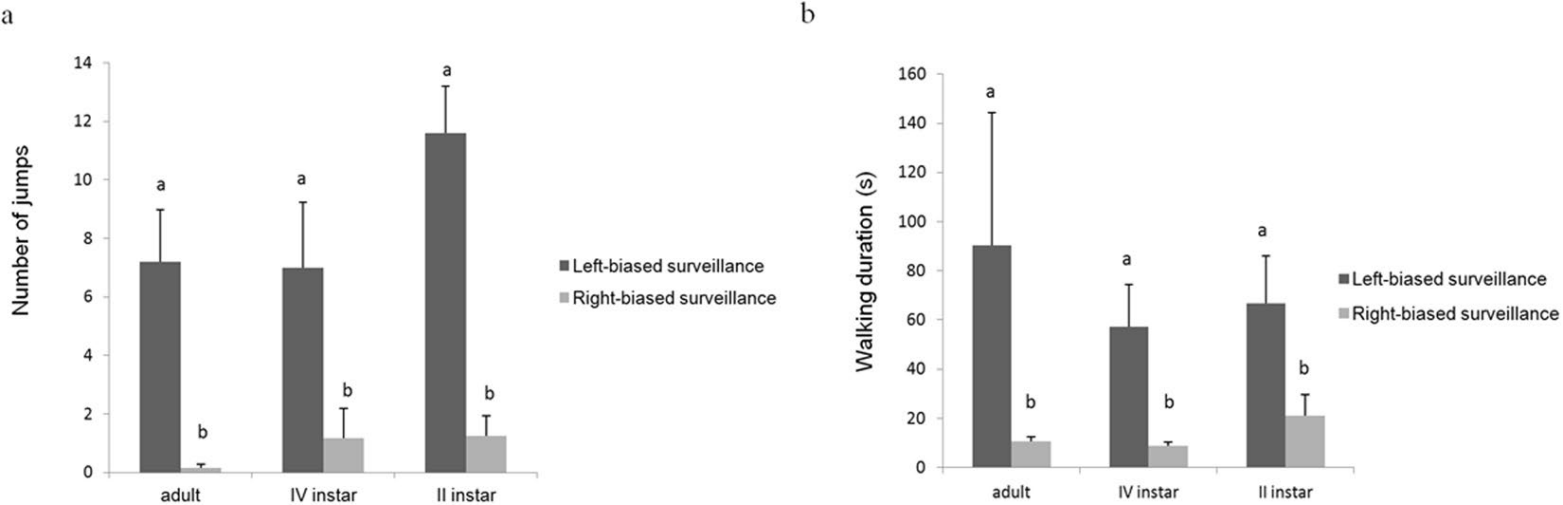
(**a**) Number of *Locusta migratoria* lateralized jumps during surveillance of a Guinea fowl-mimicking robotic predator. Different letters above each column indicate significant differences. T-bars represent standard errors. (**b**) Duration of *L. migratoria* walking during surveillance of a Guinea fowl-mimicking robotic predator. Different letters above each bar indicate significant differences. T-bars represent standard errors.

The number of locust jumps (*F*_*2,87*_ = 1.0136; *P* < 0.3672) (Fig. 3a) and the walking time spent during predator surveillance (*F*_*2,87*_ = 0.1987; *P* < 0.8253) (Fig. 3b) were not significantly affected by the tested instar. The number of jumps (*F*_*1,84*_ = 60.161; *P* < 0.0001) (Fig. 3a) and the walking time spent during predator surveillance (*F*_*1,86*_ = 30.410; *P* < 0.0001) (Fig. 3b) showed significant differences between right- and left-biased lateralized responses. Locusts with preferential use of the left eye for predator surveillance showed higher jumping and walking activity over right-biased individuals, without significant differences among developmental stages (Fig. 3).

## Discussion

Predator-prey interactions are crucial in shaping the fitness of animal species worldwide^1–4^. In several vertebrate species, lateralized processes have been reported for predator-prey interactions^5–10^, while little is known about them in arthropods. In our experiments, we investigated the lateralization of escape and surveillance responses in young instars and adults of *L. migratoria* during interactions with a biomimetic robot-predator inspired by the helmeted Guinea fowl. Results showed an individual-level lateralization in the jumping escape of *L. migratoria* exposed to a simulated predator attack. The laterality of this response increased in *L. migratoria* adults if compared to young instars.

We noted that the *L. migratoria* escape response was lateralized at individual-level in adults and IV instar individuals, although lateralization at population level was not detected. Previous studies also reported lateralization at individual level for forelimb use of *S. gregaria* ^37^, showing that stronger lateralization provides an advantage in terms of boosted motor control. Furthermore, II instar locusts did not show lateralized jumping escape responses. One of the main disadvantage of lateralization is predictability, which may be used for predation by other species or cannibalization by individuals of the same species^9,73^.

The presence of individual-level lateralization in locusts could be a strategy to avoid the cost of predictability, everyone has a different lateral bias when approached by the predator. Moreover, individual-level lateralization in the escape behaviour can contribute to the jumping performance thanks to doubled reinforcement by experience (e.g., improvement of the jumping escape performance over time centred on one of the two forelegs), as reported for other insect species^21,26,37^. The role of experience in producing stronger lateralized locusts can contribute to explain our data reporting a significant increase of lateralization strength in jumping escape from II instar to adult locusts. Indeed, locusts can exhibit motor learning at single ganglia-level^37,74^. In addition, it has been proposed that the specialization of forelimb movements control can be related to motor circuits and mechano-sensory reflexes within the prothoracic ganglion of locusts^75,76^. Lastly, we cannot exclude the effect that the post-embryonic development of the neural system can play, leading to insects with a stronger lateralization as they grow^69–72^.

Furthermore, population-level lateralization of predator surveillance was found testing both adults and young instars, showing that *L. migratoria* used the right compound eye to oversee the Guinea fowl-mimicking predator (Fig. 2c). Static cryptic behaviour of locusts overseeing the predator^30,31^ was efficiently performed by right-biased insects over left-biased ones. The latter was “more visible” to a potential predator by exhibiting a higher number of jumps and a longer walking activity. Interestingly, it has been reported that right-biased honeybees (*Apis mellifera* Linnaeus) learn better a colour stimulus compared to left-biased bees^77^, suggesting an analogy with our findings.

Locusts showed several physiological and morphological changes during the gregarious phase, reflecting a modulation of the individual’s metabolism to favour greater mobility^78^. It has been argued that lateralization at population-level is more likely to evolve in social/gregarious species^9,68^. However, concerning gregarious Orthoptera, previous studies reported only individual-level lateralization^37,79^. It should be noted that, in terms of jumping, population level laterality would not evolve in this gregarious species, since this would make the group movements more predictable than if only the single individuals had lateralized jumping performances. The population-level lateralization in the eye use in *L. migratoria* could be partially linked to the need to perform specific group tasks, such as swarm coordination^9,16^, while predictability can be avoided by individual-level lateralization of escape responses. In addition, while the surveillance is mainly accomplished by visual sense, more stimuli are involved in the escape behaviour, such as mechano-receptive hairs that sense air displacement around insects and alert them when a predator is attacking^80^. This can be related to different reactions of locusts to predators, concerning lateralized responses during surveillance and escape. Further research is needed to investigate how these lateralized traits can be connected each other in *L. migratoria*, and how they change during different social phases.

To the best of our knowledge this is the first report of lateralized predator-prey interactions in insects. Our findings outline the possibility of relying to biomimetic robotic predators to study predator-prey interactions in arthropods, avoiding the use of real predators, thus achieving highly standardized experimental conditions to investigate complex and flexible behaviours.

## Materials and Methods

### Ethic statements

This research adheres to the guidelines for the treatment of animals in behavioural research and teaching^81^. All treatments of the experimental animals complied with the laws of the country (Italy) in which the study was performed (D.M. 116192) and the European Union regulations^82^. All experiments are behavioural observations, and no permits are required in the country where the experiments are conducted. Insects were treated as gently as possible given the constraints of the experimental design. None were injured or killed during the experiments. The health of every animal was constantly assessed by checking that they fed and behaved normally.

### Locust rearing and general observations

Experiments were conducted on second-instar, fourth-instar, and adult *L. migratoria*. Locusts were maintained under controlled conditions (25 ± 1 °C, 55 ± 5% R.H., 16:8 h L:D) at the BioRobotics Institute laboratories (Scuola Superiore Sant’Anna, Pontedera). They were fed by wheat, vegetables and water^32,37^. Only animals with intact eyes, legs, wings and antennae were used for experiments.

Experiments were conducted in the laboratory (25 ± 1 °C, 55 ± 5% R.H.) during December 2016-March 2017 in a room illuminated with fluorescent daylight tubes (16:8 h L:D, lights on at 6:00). Neon tubes (Philips 30 W/33) were used; light intensity around the test arena was ca. 1000 lx, estimated over the 300–1100 nm waveband with an LI-1800 spectroradiometer (LICOR Inc., Lincoln, NE, U.S.A.), equipped with a remote cosine receptor. Directional light cues were avoided by using diffuse laboratory lighting to reduce reflection and phototaxis. For each experiment, the behaviour of locusts was directly recorded by an observer^21,27,83^. A white wall of filter paper (Whatman) surrounded both the arena and the robot, the observer was dressed in a white coat, to minimize his impact on *L. migratoria* behaviour^27,84^ and was placed symmetrically behind the robot at a reasonably distance from the arena.

### Robotic predator model

**Figure 4:**
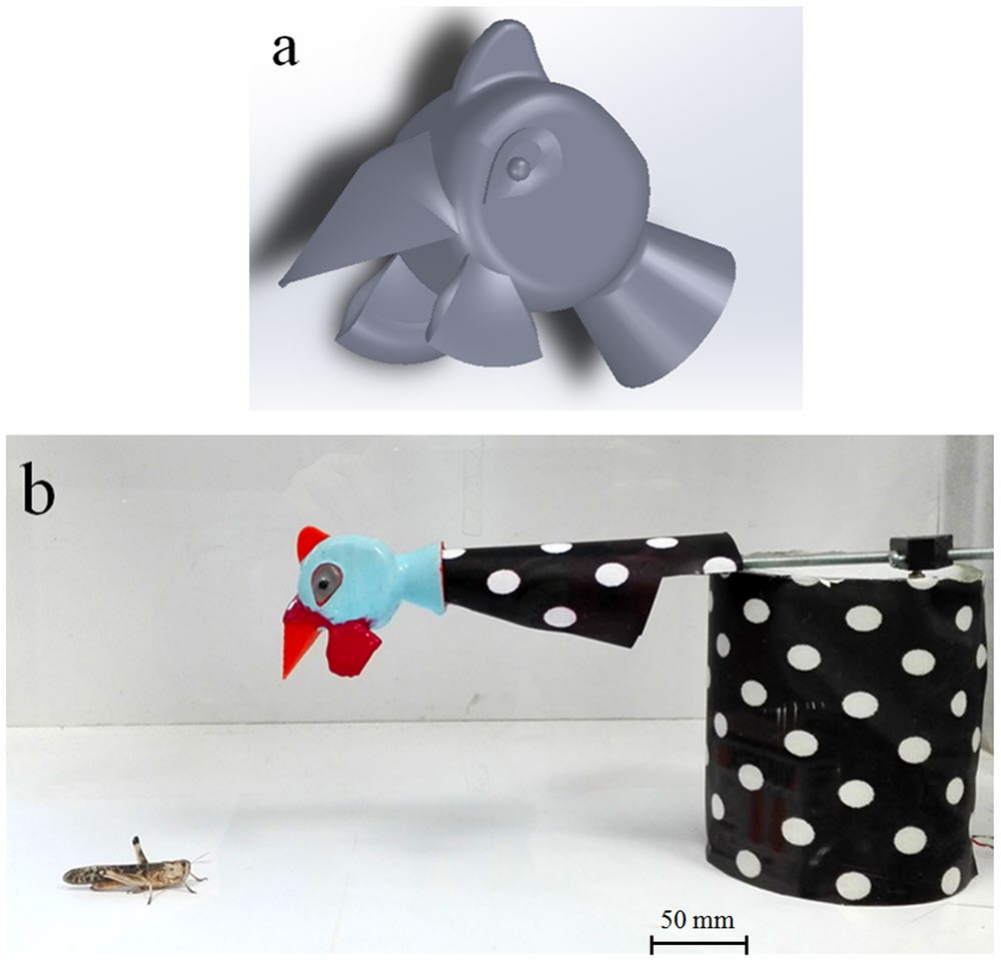
(**a**) Helmeted Guinea fowl head-replica design, and (**b**) the Guinea fowl-mimicking robotic predator with an adult locust.

To simulate a predator of locusts, a *N. meleagris* head in acrylonitrile butadiene styrene (ABS) (Fig. 4a)^38–40^ was designed in SolidWorks (Dassault Systemes, Vélizy-Villacoublay, France) and fabricated by rapid prototyping. The *N. meleagris* head-replica had a diameter of 40 mm a thickness of 30 mm and a total length, including the beak, of 75 mm. The bird head (except the beak and helmet), was covered by a thin layer of silicone rubber (Dragon Skin), by turning molding and then coloured, reproducing the colour pattern of real *N. meleagris* birds. A 300-mm steel rod, connected the bird head to a DC motor (Precision Microdrives: 225–202), producing a simple robotic arm. The DC motor was placed in a Plexiglas pipe section (height 150 mm, diameter 120 mm), partially filled with iron weights, as a support body. A grey-black sheet with white polka dots covered both the support body and partially the rod, to improve the resemblance with the *N. meleagris* plumage (Fig. 4b). A microcontroller (Arduino, Mega 2560) was used to control the movement of the robotic arm, capped with the plastic bird head. Depending on the experiment, the robotic stimulus can be moved in an upright way or horizontally by changing the support base of the pipe.

### Experiment 1: laterality of escape responses in locusts

**Figure 5:**
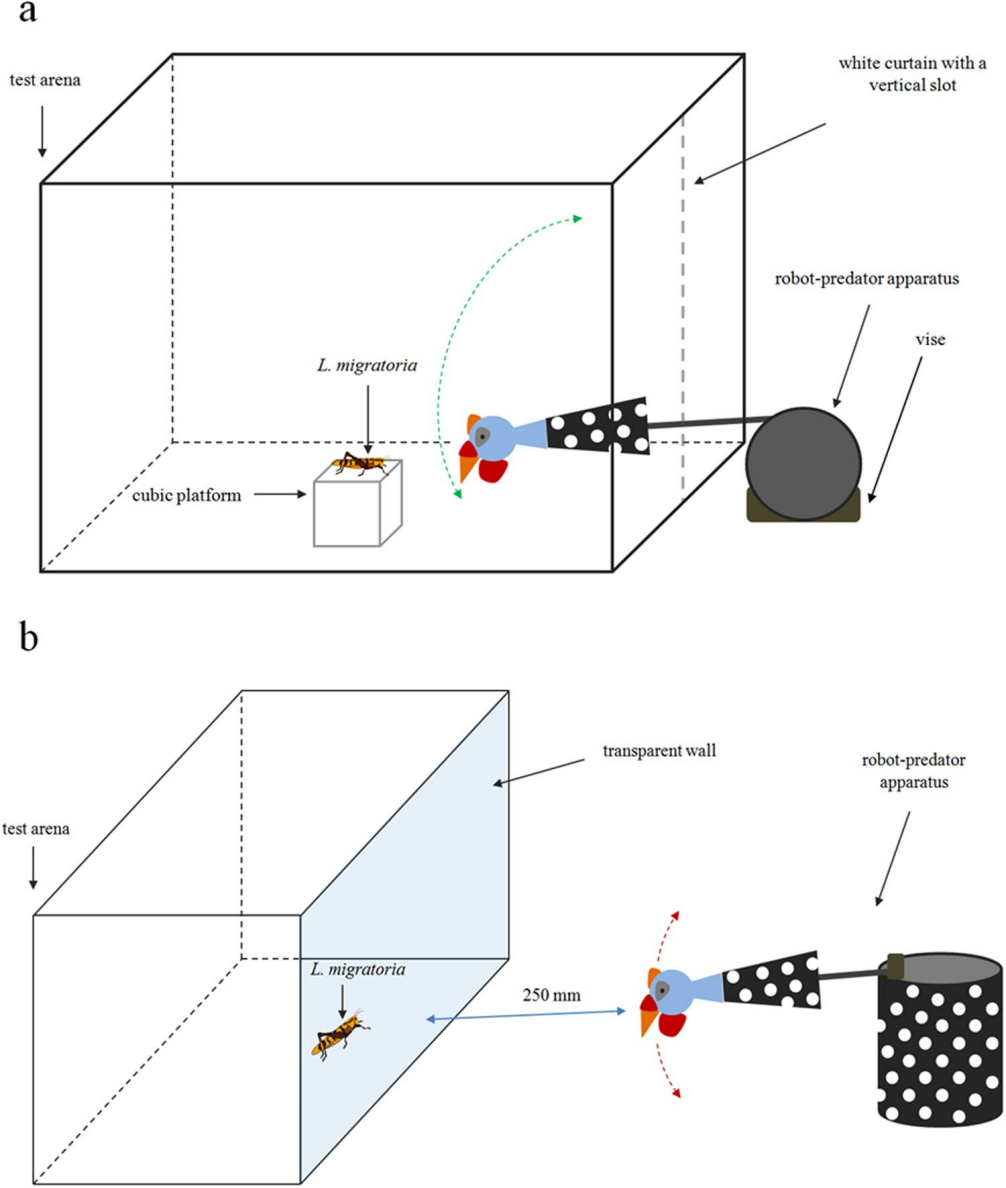
(**a**) Experiment 1 setup. The Guinea fowl-mimicking robot-predator moves vertically from top to bottom and then backing on, evoking a jumping escape of the locust. (**b**) Experiment 2 setup. The Guinea fowl-mimicking robot is yawed in front of the transparent wall of the test arena to be overseen by the locust.

We evaluated if locusts showed a bias in jumping to the left or to the right when the robot-predator approached them frontally. Individual *L. migratoria* were gently placed on a cubic platform (100 × 100 × 100 mm) of white cardboard exactly centered with respect to the robotic stimulus, in the centre of a rectangular white arena (800 × 600 × 600 mm)^37^. Insects were placed to an identical distance from the right and left side of the arena. The robotic stimulus was visually isolated from the tested subjects by a wall of the arena (600 × 600 mm) fitted with a white curtain with a vertical slot in the centre, which allowed the robotic arm, capped with the bird head, to enter in the arena when simulating predation (Fig. 5a).

A jumping escape was evoked when the robot predator simulated an attack, striking moving from top to bottom, 100 mm from the locust head, and then returning. The robot was perfectly symmetric in appearance and movement to avoid any lateral bias. The top of the arena was covered with a transparent partition, to allow focal observations of the locust behaviour. For laterality observations, we considered only locusts which were approached by the robot predator when they were perfectly centered with respect to the robot predator. For each insect, the laterality of 30 jumps was recorded. Each jump was evoked by the Guinea fowl-mimicking robot predator after 10 min from the previous one. 30 II instar, 30 IV instar and 30 adult locusts that jumped after a perfectly symmetric robotic stimulus were analyzed in this experiment.

### Experiment 2: laterality of predator surveillance in locusts

The eye preference used by locusts to scan a Guinea fowl-mimicking robotic predator was investigated. Locusts were placed individually in the centre of a rectangular white arena (800 × 600 × 600 mm), with their body axis orthogonal to a transparent wall (800 × 600 mm, Plexiglas). Insects were placed to an identical distance from the right and left side of the arena. The robot predator was placed outside the arena in correspondence of its middle, with the bird head 250 mm from the transparent wall. The rod capped with the bird head was yawed by the DC motor, 45° to the left and 45° to the right with a frequency of 0.5 Hz, to be perfectly symmetric in appearance and movement. Tests (lasting 30 minutes) started 5 minutes after the locust was introduced in the arena, removing an opaque partition from the transparent wall, that allowed visual contact with the robotic stimulus (Fig. 5b). At the beginning of the experiment, the locust was placed head on to the robotic stimulus and was able to orientate the body, according to its overseeing of the robot predator.

For each locusts, we recorded how long a given side of the insect’s body (e.g., steered body axis forming an angle >45°, with the initial orientation of the body axis perfectly centred with the stimulus, to have just one eye able to see the robot^85,86^), was exposed to the Guinea fowl-mimicking robot predator. Furthermore, since locusts assume a static pose to go unnoticed when a predator is nearby^30,31^, we recorded the number of jumps and the duration of the walking behaviour, to evaluate if the cryptic behaviour of subjects overseeing the predator was affected by lateral bias. The further distance of the robot predator and its slow movement, if compared to the Experiment 1, was predicted to evoke the cryptic behaviour in locusts over the jumping escape^30,31^. 30 II instars, 30 IV instars and 30 adults of *L. migratoria* were tested.

### Data analysis

A laterality index (LI) was calculated for each insect, to analyse the differences in the direction of jumping escape responses:

LI = [(number of jumps to the right − number of jumps to the left)/(number of jumps to the right + number of jumps to the left)]^87,88^.

We calculated a LI for each insect evaluating bias in the use of the right and left eye during predator surveillance:

LI = [(duration of surveillance with the right eye − duration of surveillance with the left eye)/(duration of surveillance with the right eye + duration of surveillance with the left eye)]^88^.

Individual asymmetrical dominance was determined by comparing the size of the LI value (ranging from −1 to + 1)^88,89^, with a threshold (LI_TH_) set to 0.3 (e.g. LI >0.3 right-biased; LI <−0.3 left-biased)^89,90^. Furthermore, the absolute value of the laterality index (ABLI) was considered, to discriminate individuals with a bilateral dominance from individuals with a lateral dominance, regardless the left or the right direction of the bias^88,89^.

Laterality differences among the numbers of locusts (II young instars, IV young instars and adults) displaying right- or left-biased jumping escapes, as well as right- or left-biased eye use during surveillance were analysed by JMP 9 (SAS) using a weighted generalized linear model (glm): *y* = *Xß* + *ε* where *y* is the vector of the observations (i.e., escape response or surveillance), *X* is the incidence matrix linking observations to fixed effects, *ß* is the vector of fixed effects (i.e., the tested instar and laterality) and *ε* is the vector of the random residual effects. A probability level of P < 0.05 was used for the significance of differences between means. Furthermore, differences in the (*i*) mean duration of walking response as well as (*ii*) the number of jumps during the surveillance of a robotic predator were analysed using the glm described above with normal distribution, fixed effects were the tested instar and laterality of the behavioural response. Averages were separated by Tukey’s HSD test. A probability level of P < 0.05 was used for the significance of differences between means.

Within each locust instar, the difference in the number of locusts using left or right eyes during the exposure to the predator was analyzed using a *χ* ^2^ test with Yates correction (P < 0.05).

## Electronic supplementary material

Dataset 1
